# YB-1 Phosphorylation at Serine 209 Inhibits Its Nuclear Translocation

**DOI:** 10.3390/ijms23010428

**Published:** 2021-12-31

**Authors:** Ekaterina M. Sogorina, Ekaterina R. Kim, Alexey V. Sorokin, Dmitry N. Lyabin, Lev P. Ovchinnikov, Daria A. Mordovkina, Irina A. Eliseeva

**Affiliations:** 1 Group of Protein Biosynthesis Regulation, Institute of Protein Research, Russian Academy of Sciences, 142290 Pushchino, Russia; kategrigoreva@vega.protres.ru (E.M.S.); ekim3@mdanderson.org (E.R.K.); asorokin@mdanderson.org (A.V.S.); lyabin@vega.protres.ru (D.N.L.); 2 Department of Leukemia, The University of Texas MD Anderson Cancer Center, Houston, TX 77030, USA; 3 Gastrointestinal Medical Oncology, The University of Texas MD Anderson Cancer Center, Houston, TX 77030, USA

**Keywords:** YB-1, Akt, nuclear transport, S102, S209, phosphorylation

## Abstract

YB-1 is a multifunctional DNA- and RNA-binding protein involved in cell proliferation, differentiation, and migration. YB-1 is a predominantly cytoplasmic protein that is transported to the nucleus in certain conditions, including DNA-damaging stress, transcription inhibition, and viral infection. In tumors, YB-1 nuclear localization correlates with high aggressiveness, multidrug resistance, and a poor prognosis. It is known that posttranslational modifications can regulate the nuclear translocation of YB-1. In particular, well-studied phosphorylation at serine 102 (S102) activates YB-1 nuclear import. Here, we report that Akt kinase phosphorylates YB-1 in vitro at serine 209 (S209), which is located in the vicinity of the YB-1 nuclear localization signal. Using phosphomimetic substitutions, we showed that S209 phosphorylation inhibits YB-1 nuclear translocation and prevents p-S102-mediated YB-1 nuclear import.

## 1. Introduction

YB-1 is a multifunctional protein involved in numerous cellular processes, including cell proliferation, differentiation, and migration (reviewed in [1,2]). By binding to DNA and RNA, it regulates mRNA transcription, alternative splicing, translation, and stability (reviewed in [3]). The functions of YB-1 depend on its subcellular localization that is dictated by the interplay between the nuclear localization signal (NLS, a.a.186–205) and the cytoplasmic retention site (CRS, a.a. 267–293) [4,5]. The YB-1 NLS is recognized by transportin 1 [6]. It is believed that the activity of the CRS prevails over the NLS, resulting in the predominantly cytoplasmic localization of YB-1 [4]. YB-1 translocates to the nucleus at the boundary of the G1/S [5] and G2/M phases [7], upon serum, IL-1β, and IFNγ stimulation [8,9,10], transcription inhibition [11,12,13], UV irradiation [14], and under oxidative stress [15]. YB-1 is over-expressed in various human cancers and its nuclear localization is associated with the multidrug-resistant phenotype, cancer progression, and a poor prognosis (reviewed in [16,17,18,19]).

YB-1 distribution between the cytoplasm and the nucleus is regulated by RNA and protein binding (reviewed in [3]), limited 20S proteasome proteolysis [20], and posttranslational modifications (reviewed in [3,21]). The most studied posttranslational modifications are the S102, S165, and S176 residues phosphorylated by Akt/RSK, casein kinase II, and casein kinase I, respectively [8,9,10,22,23,24,25]. The phosphorylation at these residues promotes YB-1 nuclear translocation [8,9,10,23,26]. On the other hand, in some cell lines, phosphorylated YB-1 does not translocate to the nucleus [4,22]. More than 10 amino acid residues located within or near the YB-1 NLS can be modified (summarized at https://www.phosphosite.org [27], v6.6.0.2, accessed on 30 December 2021) and could potentially affect YB-1 nuclear–cytoplasmic transport. Here, we report that YB-1 nuclear translocation is negatively regulated by S209 phosphorylation. This residue is located in close vicinity downstream of YB-1 NLS and is phosphorylated by Akt kinase in vitro. Moreover, using an in vitro transport assay, we demonstrated that S209 phosphorylation prevents YB-1 nuclear translocation even in the presence of phosphorylated S102.

## 2. Results

### 2.1. Serum Stimulation Does Not Induce YB-1 Nuclear Translocation in HeLa, NIH3T3, and PC-3 Cells

**Figure 1:**
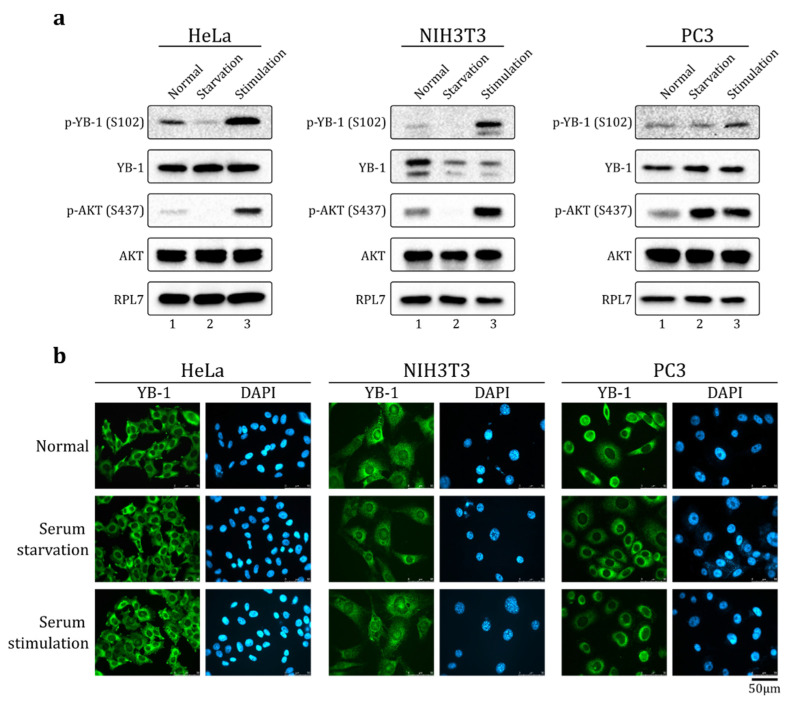
YB-1 is phosphorylated at S102 but is not translocated to the nucleus upon serum stimulation. HeLa (left panel), NIH3T3 (middle panel), and PC3 (right panel) cultivated in normal, serum starvation (24 h w/o FBS, fetal bovine serum), or serum stimulation (24 h without FBS + 2 h 20% FBS) conditions. The cells were (**a**) lysed and analyzed by Western blot using indicated antibodies, or (**b**) analyzed by immunofluorescence (IF) microscopy using antibodies against YB-1. Nuclei were visualized by DAPI staining.

It is known that S102 phosphorylation promotes YB-1 nuclear translocation [8,23]. Previously, it was shown that serum stimulation in some cell lines results in Akt activation, YB-1 S102 phosphorylation, and nuclear accumulation of YB-1 [8]. Indeed, in HeLa, NIH3T3, and PC-3 cells, the level of phosphorylated Akt kinase and S102-phosphorylated YB-1 increased upon serum stimulation (Figure 1a). However, we found that in HeLa and PC-3 cells, YB-1 did not accumulate in the nucleus (Figure 1b) and was exclusively localized to the cytoplasm in normal (10% FBS, fetal bovine serum), serum starvation (24 h w/o FBS), and serum stimulation (24 h without FBS followed by 2 h 20% FBS) conditions. In NIH3T3 cells (Figure 1b, middle panel and Figure S1), a small fraction of YB-1 was found in the nuclei and the major fraction remained in the cytoplasm, independent of culture conditions.

Therefore, there may be other YB-1 modifications that could influence YB-1 nuclear transport in these cell lines. For example, Akt kinase could phosphorylate other residues in the YB-1 molecule in addition to S102. Such modifications could inhibit YB-1 nuclear translocation mediated by S102 phosphorylation.

### 2.2. Akt Kinase Phosphorylates YB-1 at the S209 Residue In Vitro

**Figure 2:**
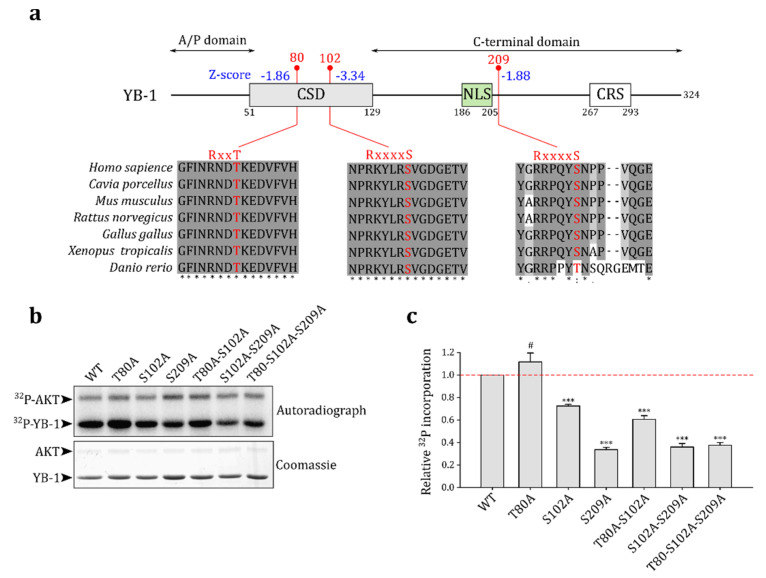
Akt phosphorylates S209 of YB-1 in vitro. (**a**) Schematic representation of predicted Akt phosphorylated sites in the YB-1 molecule (ScanSite Z-scores are indicated in blue). Positions of CSD (cold shock domain), NLS (nuclear localization signal), and CRS (cytoplasmic retention site) are shown as colored boxes. Vertebrate sequence alignments of particular segments of the YB-1 protein in the vicinity of phosphorylation sites are shown. Phosphorylation sites and Akt kinase consensus motifs are highlighted in red. (**b**) In vitro kinase assay for wild-type (WT) and mutant YB-1 proteins. The assay was performed by incubating 1 μg of YB-1 with 0.25 μg of activated Akt kinase in the assay buffer containing [γ-^32^P]-ATP. The reaction products were analyzed by SDS-PAGE (Coomassie staining, bottom panel) and autoradiography (upper panel). (**c**) Quantification of in vitro kinase assay. The YB-1 phosphorylation level was normalized to that of the WT and corrected for the corresponding protein amount determined by Coomassie staining. The mean ± SD of four independent experiments is shown. #: non-significant, *** *p* < 0.001, one-sample *t*-test.

To predict possible Akt phosphorylated sites in the YB-1 molecule, we used ScanSite 4.0 [28]. In addition to the known site S102, the program predicted two other phosphorylation sites: T80 and S209 (Figure 2a). Indeed, the phosphorylation at these sites was observed in high-throughput experiments (summarized at https://www.phosphosite.org [27], v6.6.0.2, accessed on 30 December 2021) in human and mouse cells. These residues and appropriate Akt consensus sequences are conserved among the vertebrates (Figure 2a), suggesting that they may also serve as Akt phosphorylation sites in other organisms.

We performed an in vitro Akt kinase phosphorylation assay to validate predictions using wild-type YB-1 and single, double, and triple YB-1 mutants where one, two, or three predicted Akt phosphorylation sites, respectively, were substituted by alanines (Figure 2b). The S102A and S209A mutations reduced YB-1 phosphorylation by Akt, whereas the T80A mutation slightly increased YB-1 phosphorylation. The latter could be explained by alterations in S102 accessibility to Akt in the T80A YB-1 mutant protein. The T80 residue is likely not an Akt substrate and is probably phosphorylated in vivo by other kinases.

Thus, we have confirmed that S102 and S209 indeed serve as Akt phosphorylation sites in the YB-1 molecule. Our observations are in agreement with high-throughput data for mouse adipocytes [29], where the levels of p-S102 (S100 in mice) and p-S209 (S207 in mice)-containing peptides of YB-1 increased six-fold and four-fold, respectively, upon insulin stimulation. This suggests that both S102 and S209 can be phosphorylated in vivo by insulin-activated kinases, including but not limited to Akt. In addition, ScanSite 4.0 [28] predicted that S209 in the YB-1 molecule can be phosphorylated by other kinases including phosphoinositide-dependent kinase 1, which also can be activated by insulin.

### 2.3. S209 Phosphorylation Inhibits YB-1 Nuclear Translocation

S209 is located a short distance from the NLS (Figure 2a). It is known that positively charged amino acids in the NLS are essential for its functioning [30,31]. Hence, the negative charge introduced by S209 phosphorylation could affect the YB-1 nucleocytoplasmic transport.

To verify if S209 phosphorylation affects YB-1 nuclear translocation, we used mutant HA-YB-1 proteins (S209A and S209D) that carried phosphomimetic amino acid substitutions: the S-to-A substitution imitates the dephosphorylated state, and the S-to-D substitution imitates the phosphorylated state. As controls, we used wild-type HA-YB-1 as well as S102A and S102D mutants (Figure S2).

**Figure 3:**
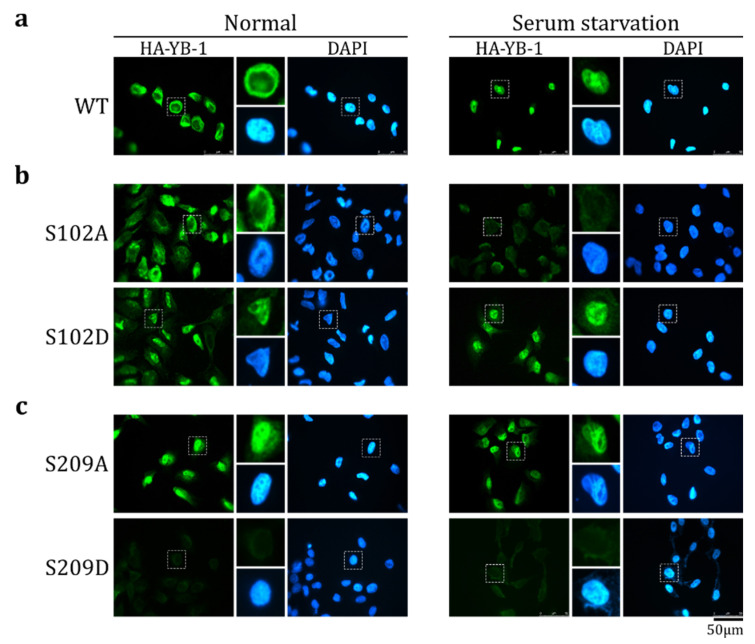
S209 phosphorylation inhibits YB-1 nuclear transport. Wild-type (WT) HA-YB-1 (**a**), HA-YB-1 S102A ((**b**), **upper** panel), HA-YB-1 S102D ((**b**), **bottom** panel), HA-YB-1 S209A (**c**), **upper** panel), and HA-YB-1 S209D ((**c**), **bottom** panel) were tested in an in vitro transport system. HeLa cells were cultivated in normal (**left** panel) and serum starvation (**right** panel) conditions before permeabilization and to obtain cytosol. Localization of HA-YB-1 was analyzed by IF microscopy using anti-HA antibodies. Quantification of at least three independent replicates of the in vitro transport assay are shown in Figure S3.

To reduce the possible influence of other YB-1 modifications on its transport in living cells, the transport was studied in an in vitro transport system. The cytosol and nuclei were prepared from cells cultivated in normal and serum starvation conditions. As was shown previously [6], HA-YB-1 was not transported to the nucleus when using the cytosol and nuclei derived from normally cultivated HeLa cells, whereas the cytosol and nuclei from serum-starved cells translocated HA-YB-1 to the nucleus in the majority of cases (Figure 3a). Of note, in the in vitro transport assay, the target proteins are either translocated to the nucleus (providing nuclear staining) or remain in the cytosol. In the latter case, they should be washed off completely and should not induce any signal upon staining. As it was shown previously [6], in normal conditions, HA-YB-1 does not translocate to the nucleus. However, it also does not wash off completely from the cytoplasm. It is known, that HA-YB-1 can be associated with actin or tubulin [32,33], which may explain its cytoplasmic staining after washes.

HA-YB-1 S102A was not transported to the nucleus in both normal and serum starvation conditions (Figure 3b, upper panel). On the contrary, the S102D mutant was efficiently translocated to the nucleus in both conditions (Figure 3b, bottom panel). Therefore, we concluded that S102 phosphorylation positively regulates YB-1 nuclear translocation, which is in good agreement with the previous cell-based assays [8,23,26].

HA-YB-1 S209A, a dephosphorylation mimetic, translocated to the nucleus in both normal and serum starvation conditions (Figure 3c, upper panel), whereas phosphomimetic HA-YB-1 S209D was not transported in any studied conditions (Figure 3c, bottom panel). Thus, we concluded that S209 phosphorylation inhibits YB-1 transport. Interestingly, HA-YB-1 S209D, in contrast to wild type HA-YB-1, is completely washed off and does not remain in the cytoplasm after transport reaction. We speculate that it could be caused by altered binding of the S209D mutant to the cytoskeleton.

### 2.4. S209 Phosphorylation Does Not Change the RNA-Binding Ability of YB-1

It is known that YB-1 can stay in the cytoplasm through RNA binding [11]. The mutations of the RNP (ribonucleoprotein) consensus motifs in the cold shock domain decrease YB-1-to-RNA binding affinity and YB-1 nucleus translocation [4,34,35]. In addition to the CSD, positively charged amino acid residue clusters in the C-terminal domain of YB-1 also contribute to its RNA binding [3,36,37,38]. S209 is located in the vicinity of a positively charged cluster, and its negative charge upon phosphorylation could reduce the YB-1-to-RNA binding. To test this hypothesis, we performed EMSA with wild-type YB-1 and S102 and S209 mutants.

**Figure 4:**
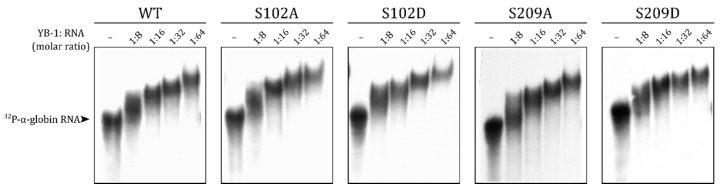
S102 and S209 phosphomimetic substitutions do not affect the YB-1-to-RNA binding. WT and mutant YB-1 binding to nonspecific [^32^P]-labeled 660-nt α-globin RNA was analyzed with EMSA.

In agreement with previous observations [22], the S102 mutant bound to RNA with the same efficiency as the wild-type protein (Figure 4). Mutations of S209 also did not affect the YB-1-to-RNA binding (Figure 4). Therefore, the observed differences in the nuclear transport of HA-YB-1 mutants were not caused by changes in the RNA binding affinity. 

### 2.5. The Effect of S209 Phosphorylation on YB-1 Transport Prevails over That of S102 Phosphorylation

The phosphorylation of S102 and S209 results in opposite effects on YB-1 translocation, and both residues can be phosphorylated simultaneously [29]. It is of interest to reveal the combined effect of both modifications on YB-1 transport.

**Figure 5:**
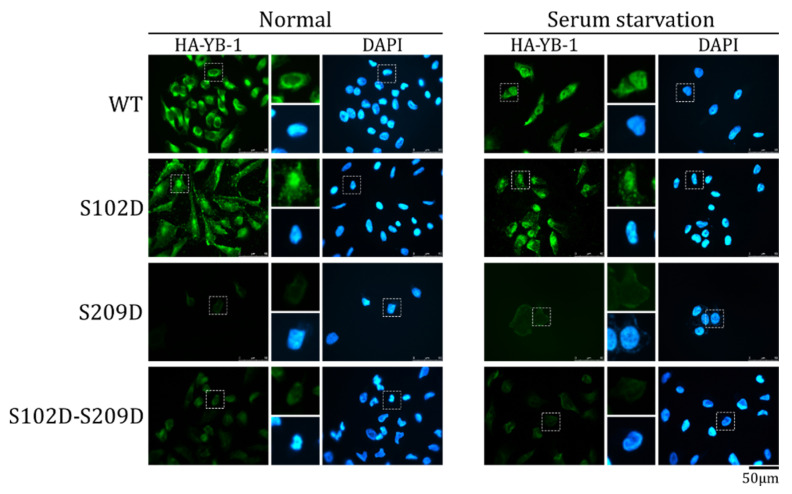
S209 phosphorylation inhibits S102-dependent YB-1 nuclear transport. Wild type (WT) HA-YB-1, HA-YB-1 S102D, HA-YB-1 S209D, and HA-YB-1 S102D-S209D recombinant proteins were used in an in vitro transport system. HeLa cells were cultivated in normal (**left** panel) and serum starvation (**right** panel) conditions before permeabilization and to obtain cytosol. Localization of HA-YB-1 was analyzed by IF microscopy using anti-HA antibodies. Quantification of at least three independent replicates of the in vitro transport assay are shown in Figure S3.

In contrast to HA-YB-1 S102D, a double HA-YB-1 mutant (S102D-S209D) imitating phosphorylation at both residues was not detected in the nucleus in both normal and serum starvation conditions (Figure 5). In other words, the HA-YB-1 double mutant has the S209 phosphorylation phenotype and does not demonstrate S102D behavior. We concluded that, at least in vitro, S209 phosphorylation inhibits S102 phosphorylation-dependent YB-1 nuclear translocation.

## 3. Discussion

YB-1 is a nuclear-cytoplasmic shuttling protein with functions that depend on subcellular localization. Despite the clinical significance [16,17,18,19], the mechanisms regulating YB-1 localization have not been studied in detail. More than 10% of YB-1 residues could be post-translationally modified. Among them, there are approximately 15 residues located within or near the NLS (summarized at https://www.phosphosite.org [27], v6.6.0.2, accessed on 30 December 2021); their modifications may potentially affect YB-1 nuclear import. We have found that S209 phosphorylation prevents YB-1 nuclear translocation in vitro. S209 is located near the NLS, and its post-phosphorylation negative charge could modulate RNA or protein binding. However, as we demonstrated, YB-1-to-RNA binding is not affected by S209 phosphorylation in vitro.

It is known that long noncoding RNAs (lncRNAs) regulate YB-1 localization [39,40,41,42,43]. We cannot exclude that in vivo S209 phosphorylation could alter YB-1 binding to a specific lncRNA (e.g., if stabilized by protein partners). On the other hand, S209 phosphorylation could affect YB-1 protein–protein interactions. In specific, it could destabilize the binding of transportin 1 that recognizes the NLS and transfers YB-1 to the nucleus [6]. Additionally, S209 phosphorylation may change the YB-1 binding to YB-1 NLS-recognizing proteins, such as those promoting nuclear localization (p53, DACH1, DHX9, hnRNPM, etc.) [13,44,45] or retention in the cytoplasm (HSP60, C1QBP) [34,46].

Interestingly, in addition to phosphorylation, the S209 residue of YB-1 can be O-linked β-N-acetylglucosamine modified (O-GlcNAcylated) [47]. It is known that phosphorylation and O-GlcNAcylation can have the opposite effects on the protein transport. For example, phosphorylation at S257 stimulates nuclear translocation of ZO-2 protein, whereas O-GlcNAcylation at the same residue stimulates its export from the nucleus [48]. HnRNP A1 O-GlcNAcylation enhances its binding to transportin 1 and promotes nuclear accumulation, whereas phosphorylation has the opposite effect [49]. Thus, S209 phosphorylation can influence the YB-1 transport not only directly but also by preventing O-GlcNAcylation of this residue. Moreover, it is known that O-GlcNAcylation can affect nearby phosphorylation [47,50,51]. We speculate that eliminating O-GlcNAcylation by phosphorylation at S209 may change the phosphorylation of other residues in the vicinity of the NLS.

To summarize, we have demonstrated that S209 localized near the YB-1 NLS is phosphorylated by Akt kinase and inhibits YB-1 nuclear translocation in vitro. Moreover, it can inhibit p-S102-mediated YB-1 nuclear transport. We propose that S209 phosphorylation may directly alter YB-1 protein–protein interactions or do so indirectly by eliminating O-GlcNAcylation of this residue. O-GlcNAcylation, in turn, probably affects YB-1 modifications and YB-1 protein–protein interactions. Future studies are necessary to decipher the exact mechanism and its crosstalk with p-S102-mediated YB-1 nuclear transport.

## 4. Materials and Methods

### 4.1. Expression Constructs and Recombinant Proteins

YB-1 and HA-YB-1-encoding plasmids pET-3-1-YB-1 (WT), pET-3-1-HA-YB-1 (WT), and pcDNA3.1 HA-YB-1 (WT) were described previously [6,52].

Point mutations were introduced by two-step PCR (Table 1). The resulting cDNA was cloned into pET-3-1-HA-YB-1 (WT) by *Xma*I (internal site in YB-1 coding region) and *BamH*I sites, and into pET-3-1 and pcDN3.1-HA by *Nde*I and *BamH*I sites.

Recombinant proteins were expressed in *Escherichia coli* and purified as described previously [52].

### 4.2. In Vitro Kinase Assay

The phosphorylation reaction was performed using 1 μg of YB-1 recombinant proteins and 0.25 μg (0.8 unit) of activated Akt-kinase (Upstate Biotechnology, Lake Placid, NY, USA) in a final volume of 20 μL containing 25 mM Tris-HCl (pH 7.6), 10 mM MgCl_2_, 2 mM DTT, 5% (*v*/*v*) glycerol, 25 μM ATP, and 5 μCi [γ-^32^P]ATP (4000 Ci/mM, IBCh, Moscow, Russia). After a 2 h incubation at 30 °C, the reaction products were resolved by 15% SDS/PAGE, stained with Coomassie brilliant blue R-250, and visualized by autoradiography. The relative radioactivity was determined using a Packard Cyclone Storage Phosphor System (Packard Instrument Company, Inc., PerkinElmer, Waltham, MA, USA).

### 4.3. Electrophoretic Mobility Shift Assay (EMSA)

The [^32^P]-labeled uncapped and non-polyadenylated 660 nt α-globin mRNA was transcribed from the pET28-α-globin plasmid linearized with *BamH*I by T7 polymerase in the presence of [α-^32^P]ATP (2000 Ci/mM, IBCh, Moscow, Russia), as described previously [53].

The reaction mixture contained [^32^P]-labeled α-globin mRNA and increasing amounts of YB-1 proteins (0, 8, 16, 32, or 64-fold molar excess) to a final volume of 10 μL in reaction buffer (100 mM NaCl, 20 mM Tris-HCl (pH 7.6), 0.1 μg/mL BSA, and 0.5 mM DTT). The mixture was incubated for 15 min at 30 °C. The RNA–protein complexes were separated in native 3.8% PAAG in 0.5× TBE (44.5 mM Tris, 44.5 mM boric acid, and 10 mM EDTA) and visualized by autoradiography. The relative radioactivity was determined using a Packard Cyclone Storage Phosphor System (Packard Instrument Company, Inc., PerkinElmer, Waltham, MA, USA), followed by autoradiography.

### 4.4. Cell Cultivation and Transfection

NIH3T3, HeLa, and PC3 cells (originally obtained from ATCC, American Type Culture Collection) were kindly provided by Dr. Elena Nadezhdina (Institute of Protein Research, Russian Academy of Sciences, Pushchino, Russia). The cells were cultivated in DMEM (Dulbecco’s Modified Eagle Medium), DMEM/F12 (Dulbecco’s Modified Eagle Medium/Nutrient Mixture F-12), and RPMI (Roswell Park Memorial Institute Medium), respectively. The media were supplemented with 10% fetal bovine serum (FBS, Capricorn Scientific, Ebsdorfergrund, Germany), 2 mM glutamine, and 1× Antibiotic-Antimycotic (Gibco, Waltham, MA, USA). The cells were kept at 37 °C in a humidified atmosphere containing 5% CO2. The cells were cultivated by standard methods.

For serum starvation, cells were incubated for 24 h in the appropriate medium without FBS. For serum stimulation, after 24 h of serum starvation, the cells were incubated for 2 h in medium supplemented with 20% FBS.

Transient transfection of cells was performed using Lipofectamine 3000 reagent (Invitrogen, Waltham, MA, USA) according to the manufacturer’s recommendations.

### 4.5. Western Blot

Cells were rinsed two times with phosphate-buffered saline and lysed in SDS electrophoresis sample buffer. Proteins were separated by SDS-PAGE and transferred onto a nitrocellulose membrane. The membrane was blocked with 5% nonfat milk or 7.5% BSA (for phospho-protein detection) in TBS-T (10 mM Tris-HCl, pH 7.6, 150 mM NaCl, and 0.05% Tween 20) and incubated overnight at 4 °C in TBS-T supplemented with BSA (7.5%) and appropriate antibodies. The membrane was washed three times with TBS-T, incubated for 1 h with horseradish peroxidase-conjugated goat anti-rabbit IgG (1:4000, 7074, Cell Signaling Technology, Danvers, MA, USA) in 5% nonfat milk in TBS-T, and then washed three times with TBS-T. Immunocomplexes were detected using an ECL Prime kit (GE Healthcare, Chicago, IL, USA) according to the manufacturer’s recommendations.

The rabbit primary antibodies were from Bethyl (YB-1, A303-230A, Montgomery, TX, USA), Sigma (RPL7, SAB4502656, Merk, Kenilworth, NJ, USA), and Cell Signaling Technology (Danvers, MA, USA): phospho-YB-1 S102 (C34A2), Akt (C67E7), phospho-Akt S473 (D9E). YB-1, S6, and RPL7 antibodies were used as a 1:10,000 dilution; other primary antibodies were used as a 1:2000 dilution.

### 4.6. In Vitro Transport Assay

The in vitro transport assay was performed as described previously [6]. Briefly, cytosol was prepared from HeLa cells cultivated under normal or serum starvation conditions. HeLa cells were cultivated on coverslips, permeabilized with 50 µg/mL digitonin, and soluble cytoplasm proteins were washed off with import buffer (20 mM НЕРЕS-KOH (PH 7.3), 110 mM CH_3_COOK, and 2 mM (CH_3_COO)_2_Mg). The coverslips were inverted over drops containing 5 mM creatine phosphate, 0.1 mg/mL creatine phosphokinase, 0.5 mM ATP, 0.5 mM GTP, 50% HeLa cytosol, and 50 µg/mL of target protein. The reaction mixture was incubated at 30 °C for 30 min, washed with import buffer, and analyzed by immunofluorescence microscopy using anti-HA antibodies to visualize proteins of interest.

### 4.7. Immunofluorescence Microscopy (IF)

Cells were grown on coverslips, fixed with 4% paraformaldehyde in PBS for 30 min at RT (room temperature), washed 3 times with PBS (10 min, RT), and permeabilized in PBS supplemented with 0.5% Triton X-100 for 30 min at RT. After permeabilization, the coverslips were rinsed with PBS, blocked with 5% FBS in TBS-T for 30 min at RT, and incubated with primary antibodies in blocking solution overnight at 4 °C. Then, the coverslips were washed three times with TBS-T, after which appropriate secondary antibodies in blocking solution were added for 1 h at RT. After final washes with TBS-T, the samples were assembled using ProLong Gold antifade reagent with DAPI (Molecular Probes, Waltham, MA, USA) and analyzed using a Leica TCS SPE fluorescence microscope (Wetzlar, Germany).

The primary antibodies, rabbit anti-YB-1 (A303-230A, Bethyl, Montgomery, TX, USA), and mouse anti-HA (H9658, Sigma, Merk, Kenilworth, NJ, USA) were used as 1:2500 dilution. The secondary Alexa 488-linked antibodies, anti-rabbit IgG (A11008, Invitrogen, Waltham, MA, USA), and anti-mouse IgG (A11001, Invitrogen, Waltham, MA, USA) were used as a 1:1000 dilution.

### 4.8. Phosphorylation Site Predictions and Conservation Analysis

The Akt phosphorylation sites in the YB-1 molecule were predicted using ScanSite 4.0 [28]. For sequence alignment, protein sequences of YB-1 orthologs were extracted from OrthoDB v 10.1 [54] and aligned using ClustalW [55].

## Figures and Tables

**Table 1:** List of primers. The nucleotides introducing corresponding mutations are in bold; the sites of restriction endonucleases are underlined.

Primer name	Sequence	Comments
For T80A	5′-CATCTTCCTT**GGC**GTCATTCCTG-3′	1st step PCR forward primers
For T80D	5′-CATCTTCCTT**GAC**GTCATTCCTG-3′	
For S102A	5′-GTACCTTCGC**GCT**GTAGGAGATGGAG-3′	
For S102D	5′-GTACCTTCGC**GAC**GTAGGAGATGGAG-3′	
For S209A	5′-CCACAGTAT**GCT**AACCCTCCTG-3′	
For S209D	5′-CCACAGTAT**GAC**AACCCTCCTG-3′	
For_NdeI	5′-ATATACATATGAGCAGCGAGGCCGAGAC-3′	2nd step PCR forward primer, *Nde*I site is underlined
Rev_BamHI	5′-TCTTCGGATCCAATCTTTTGTTCATTTC-3′	1st and 2nd steps PCR reverse primer, *BamH*I site is underlined

## Supplementary Materials

The following are available online at https://www.mdpi.com/article/10.3390/ijms23010428/s1.
