# Supplementary material for: YB-1 Phosphorylation at Serine 209 Inhibits Its Nuclear Translocation
Source: Int J Mol Sci. 2021 Dec 31;23(1):428. doi: 10.3390/ijms23010428 (PMC8745666; doi:10.3390/ijms23010428)
Supplement: Supplementary file 1 [file ijms-23-00428-s001.zip › ijms-1500120-supplementary.pdf]

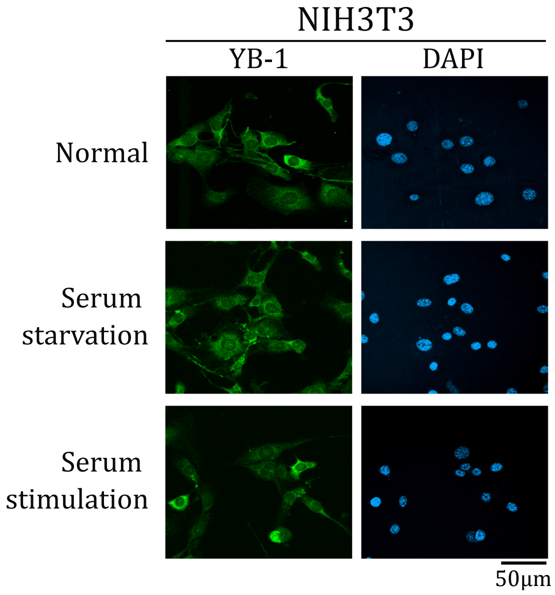

Figure S1. The confocal microscopy of NIH3T3 cells, the same coverslips as in Figure 1b. NIH3T3 cultivated in normal, serum starvation (24 h w/o FBS), or serum stimulation (24 h w/o FBS + 2 h 20% FBS) conditions. The cells were analyzed by IF confocal microscopy (Leica TCS SP5) using antibodies against YB-1. Nuclei were visualized by DAPI staining.

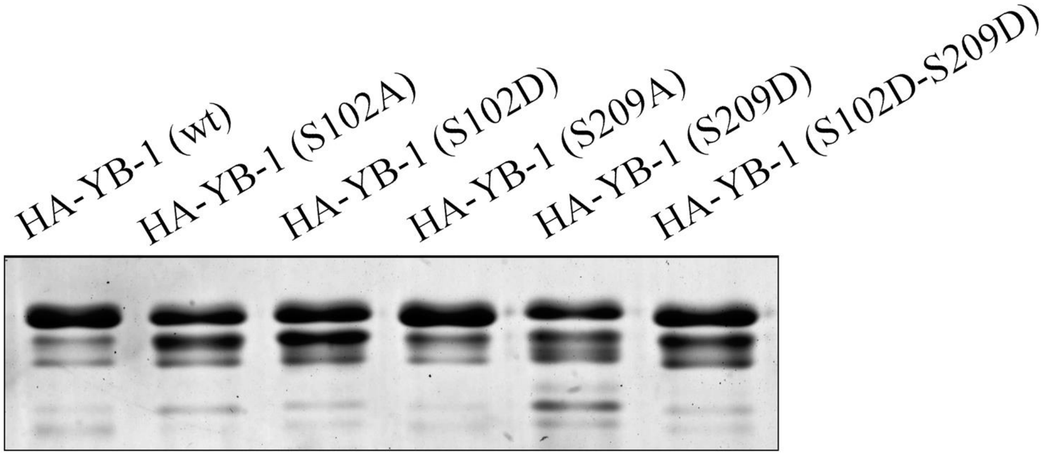

Figure S2. Coomassie-stained SDS-PAAG of HA-YB-1 proteins used in the experiments. 1 μg of HA-YB-1 proteins were resolved in 15% SDS-PAAG and stained with Coomassie R-250.

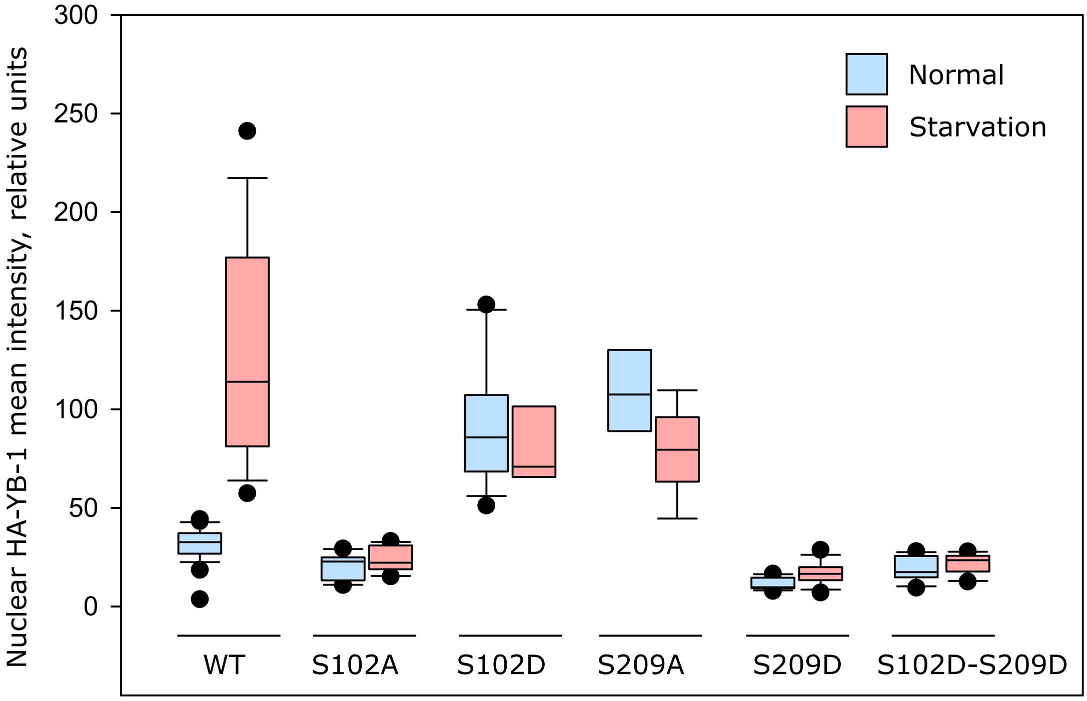

Figure S3. Quantification of results of the *in vitro* transport assay (related to Figures 3 and 5). The graphs depict the intranuclear mean Alexa488 fluorescence intensity of HA-YB-1 proteins. The Image J software was used for quantification (30-100 cells in each experiment).
